# Loss of *Pkd1* limits susceptibility to colitis and colorectal cancer

**DOI:** 10.1038/s41389-023-00486-y

**Published:** 2023-08-05

**Authors:** Anna S. Nikonova, Alexander Y. Deneka, Flaviane N. Silva, Shabnam Pirestani, Rossella Tricarico, Anna A. Kiseleva, Yan Zhou, Emmanuelle Nicolas, Douglas B. Flieder, Sergei I. Grivennikov, Erica A. Golemis

**Affiliations:** 1 Program in Cancer Signaling and Microenvironment, Fox Chase Cancer Center, Philadelphia, PA USA; 2 Molecular & Cell Biology & Genetics (MCBG) Program, Drexel University College of Medicine, Philadelphia, PA USA; 3 Department of Biology and Biotechnology, University of Pavia, Pavia, Italy; 4 Department of Pathology, Fox Chase Cancer Center, Philadelphia, PA USA; 5 Departments of Medicine and Biomedical Science, Cedars-Sinai Medical Center, Los Angeles, CA USA; 6 Department of Cancer and Cellular Biology, Lewis Katz School of Medicine at Temple University, Philadelphia, PA USA

**Keywords:** Colorectal cancer, Tight junctions

## Abstract

Colorectal cancer (CRC) is one of the most common cancers, with an annual incidence of ~135,000 in the US, associated with ~50,000 deaths. Autosomal dominant polycystic kidney disease (ADPKD), associated with mutations disabling the *PKD1* gene, affects as many as 1 in 1000. Intriguingly, some studies have suggested that individuals with germline mutations in *PKD1* have reduced incidence of CRC, suggesting a genetic modifier function. Using mouse models, we here establish that loss of *Pkd1* greatly reduces CRC incidence and tumor growth induced by loss of the tumor suppressor *Apc*. Growth of *Pkd1*^−/−^*;Apc*^−/−^ organoids was reduced relative to *Apc*^−/−^ organoids, indicating a cancer cell-intrinsic activity, even though *Pkd1* loss enhanced activity of pro-oncogenic signaling pathways. Notably, *Pkd1* loss increased colon barrier function, with *Pkd1*-deficient animals resistant to DSS-induced colitis, associated with upregulation of claudins that decrease permeability, and reduced T cell infiltration. Notably, *Pkd1* loss caused greater sensitivity to activation of CFTR, a tumor suppressor in CRC, paralleling signaling relations in ADPKD. Overall, these data and other data suggest germline and somatic mutations in *PKD1* may influence incidence, presentation, and treatment response in human CRC and other pathologies involving the colon.

## Introduction

Colorectal cancer (CRC) is one of the most common cancers, with an annual incidence of ~135,000 in the US, associated with ~50,000 deaths [1]. For the most common forms of CRC, mutational loss of the Adenomatous Polyposis Coli (*APC*) tumor suppressor gene, leading to WNT/β-catenin (CTNNB1) pathway activation, is followed by mutations activating *KRAS* and inactivating *TP53* during progression [2, 3]. A number of modifier genes have been recognized as influencing CRC risk, tumor aggressiveness and response to therapy, and overall survival [4, 5]. For example, individuals with familial adenomatous polyposis (FAP), associated with truncating germline mutations in *APC*, or with Lynch syndrome, associated with germline mutations in DNA mismatch repair (MMR) genes, experience a higher incidence of CRC, and disease onset at an earlier age [6]. It is estimated that there are over 100 inherited gene variants modifying CRC risk [7].

This study was motivated by results from two studies investigating genes associated with a genetically inherited developmental syndrome. Autosomal dominant polycystic kidney disease (ADPKD, MIM #173900; # 613095) typically arises from germline mutations inactivating the *PKD1* or *PKD2* genes, and affects approximately 1 in 1000 individuals, and has a phenotype of epithelial cell overgrowth, resulting in extensive replacement of normal renal ductal and tubular structure with fluid-filled cysts. In a study comparing 10,146 kidney recipients with ADPKD, versus 107 339 kidney recipients without ADPKD, individuals with ADPKD had a reduced incidence of CRC (incidence rate ratio 0.72), as well as lower incidence of other cancers affecting the gastrointestinal tract [8]. Although this is a modest signal, it was obtained in a population where ~15% of the individuals were likely to have mutations in *PKD2*, associated with a mild disease course, and at least 30–40% of the individuals with *PKD1* mutations would have inherited a single moderately hypomorphic allele rather than a homozygous or strongly inactivating mutation [9, 10]. Independently, a study investigating *PKHD1* (mutated in autosomal recessive PKD (ARPKD), and encoding a protein, fibrocystin, which functionally interacts with PKD1), also found a lower than expected rate of CRC in mutation bearers (0.42% in 3603 controls versus 0.027% in 3767 patients with CRC; *p* = 0.0002) [11].

Although investigations of *PKD1*, *PKD2*, and *PKHD1* typically focus on their roles in the kidney, ADPKD, and ARPKD have many extrarenal manifestations [12]. The PKD1 protein is expressed and active in many tissues, including the colonic epithelium [13]. Relevant to CRC, mutations in *PKD1* have been shown to modulate the activity of the WNT/CTNNB1 and other pro-growth signaling pathways in the kidney, although the mechanism of interaction is not incompletely understood, and distinct assay systems have yielded conflicting results [14, 15]. Interestingly, in an analysis of 190 clinical CRC specimens, PKD1 was overexpressed in tumors relative to normal tissue, regardless of TNM stage, suggesting elevation at an early stage of tumorigenesis. PKD1 expression was highest in tissues with the deepest invasion and associated with poor survival [16]. Overexpression of PKD1 in CRC cell lines increased growth rate and epithelial-mesenchymal transition (EMT), enhancing migration and invasion [16]; whereas a mouse model null for the *Pkd1* gene was embryonically lethal due to a cell migration defect [17].

Based on these suggestive studies, we have used mouse models to directly test the idea that genetic loss of *Pkd1* reduces the risk of CRC arising from mutations in *Apc*, and if so, to explore relevant mechanisms. Based on this work, we define *Pkd1* loss as tumor suppressive for CRC, based on cell-intrinsic activity in the colon epithelium. We further show that *Pkd1* loss does not reduce CRC growth by inhibiting pro-proliferative pathways, but does reprogram cell-cell contacts in the colon epithelium so as to limit tissue damage and inflammation-associated proliferation.

## Materials and methods

### Mouse strains and drug treatments

The Institutional Animal Care and Use Committee (IACUC) of Fox Chase Cancer Center approved all experiments involving mice. To study DSS-induced colitis, we used tamoxifen-regulated-Cre^+^ expressed from the β-actin promoter in all tissues, crossed to *Pkd1*^*fl/fl*^ [18, 19] mice, as in [20–22], with mice referred to as *Pkd1*^−/−^ post-tamoxifen treatment. Mice lacking Cre (*Pkd1*^*fl/fl*^, referred to as Pkd^+/+^) were used as negative controls. Mice were injected intraperitoneally with tamoxifen (250 mg/kg body weight [BW], formulated in corn oil) on post-natal days P35 and P36 to induce loss of *Pkd1*. 10 weeks after injection, mice were treated with 2.5% dextran sodium sulfate (DSS) in drinking water for 5 days to induce acute colitis, and euthanized 2 days later. For some experiments, a FITC-dextran tracer (46944, Sigma-Aldrich, St. Louis, MO; 4 kDa, 0.6 mg/g body weight) was administered by oral gavage 3 hours prior to euthanasia. 300–400 μl of blood was collected immediately prior to euthanasia, and concentration of FITC-dextran was measured in the blood using a ProXpress Visible-UV-fluorescence 16-bit scanner (Perkin-Elmer, Waltham, MA). Colon tissues were collected for subsequent analysis.

To evaluate the role of *Pkd1* in CRC, *Pkd1*^*fl/fl*^ mice were crossed to *Cdx-ERT-Cre*^*+*^ mice bearing floxed *Apc* (*Cdx2-ERT-Cre*^*+*^*;Apc*^*fl/fl*^ [23–25], with colon epithelium-preferential *Cdx2* transgene expression, referred to as Apc^−/−^ post-tamoxifen treatment), to generate *Cdx2-ERT-Cre*^*+*^*;Apc*^*fl/fl*^*;Pkd1*^*fl/wt*^ and *Cdx2-ERT-Cre*^*+*^*;Apc*^*fl/fl*^*;Pkd1*^*fl/fl*^ mice (designated *Apc*^−/−^*;Pkd1*^*+/−*^ and *Apc*^−/−^*;Pkd1*^−/−^*,* respectively). These mice and control *Apc*^*fl/fl*^ mice were injected intraperitoneally with tamoxifen [250 mg/kg body weight (BW),] at the age of 3 months to induce deletion of *Apc* and *Pkd1* in the colon. Mice were euthanized 5 weeks later and colons collected for analysis.

For drug treatment experiments, inactivation of the *Apc* and *Pkd1* genes was induced in 3 months old *Cdx2-ERT-Cre*^+^;Apc^fl/fl^ and *Cdx2-ERT-Cre*^*+*^;*Apc*^*fl/fl*^*;Pkd1*^*fl/fl*^ mice. One week after tamoxifen injection, 3–4 mice of each genotype were randomly assigned to one of the following treatment groups: (1) Vehicle, (2) 5-FU (50 mg/kg) + LV (leucovorin, 90 mg/kg) + Irinotecan (24 mg/kg) – FOLFIRI, and (3) 5-FU (50 mg/kg) + LV (leucovorin 90 mg/kg) + Oxaliplatin (6 mg/kg) – FOLFOX. Drugs were administered once a week for 4 weeks intravenously, after which mice were euthanized and organs were collected for analysis.

### qRT-PCR analysis of *Pkd1* expression

Assessment of effective Cre targeting of *Pkd1* is complicated by the presence of multiple *Pkd1* pseudo-genes. We performed qRT-PCR probing for Pkd1 exon 1-2 (intact *Pkd1*) and exon 1-5 (deleted exons 2-3-4 in *Pkd1* floxed mice) junctions. The oligonucleotide sequences used for PCR are as follows: CTGCCGCGTCAATTGCT; CCTATGTCCAGCGTCTGAAGTA (Pkd1 junction 1-2). CTGCCGCGTCAATTGCT; GCAGGGAGGAAGTAATATGGAAG (Pkd1 junction 1-5). Briefly, total RNA was isolated from tumor tissues or organoids using a Zymo Research Quick-RNA MicroPrep Kit (#R1050) and tested for quality on a Bioanalyzer (Agilent Technologies, Santa Clara, CA). RNA concentrations were determined with a NanoDrop spectrophotometer (Thermo Fisher Scientific, Waltham, MA). Total RNA prepared from tumor tissue was used for quantitative RT-PCR, with expression of sequences of interest normalized to that of the housekeeping gene 36B4.

### Organoid isolation and culture

Organoid culturing was performed as in [26, 27]. Briefly, dissected colon lesions (~2–3 mm) were sequentially washed with cold PBS and cold chelation buffer on ice. Tissue was then incubated in digestion buffer (DMEM medium with 2.5% FBS, 1% collagenase, and 0.125% dispase) at 37 °C for 45 min followed by treatment with 1x accutase (#07922, StemCell Technologies, Vancouver, BC). Single cells were collected by centrifugation, resuspended in PBS, and embedded in growth factor-reduced Matrigel (CLS354230, Corning, Sigma-Aldrich, St. Louis, MO) and seeded in 24-well plates in culture medium (advanced DMEM/F12 (ADF) supplemented with penicillin/streptomycin, 10 mmol/L HEPES, Glutamax, 1x N2, 1x B27 (all from Invitroge]) and murine EGF (50 μg/ml, SRP3196, Sigma-Aldrich, St. Louis, MO). Medium was changed every 4 days during experiments.

For drug treatment experiments, organoids were treated with vehicle, a mixture of 10 μM lumacaftor + 3 μM ivacaftor, or 0.5 μM PRI-724 for 10 days. Alternatively, organoids were treated with vehicle, a mixture of 5-FU (9.6 μM) + LV (4.7 μM) + irinotecan (1 μM) – FOLFIRI, or 5-FU (9.6 μM) + LV (4.7 μM) + oxaliplatin (0.4 μM) – FOLFOX for 10 days. All drugs were purchased from MedChemExpress (Monmouth Junction, NJ). Cell or organoid viability was assessed by a CellTiterGlo luminescent cell viability assay (#G7570, Promega, Madison, WI, USA), using the manufacturer’s protocols.

### Tissue preparation, histology, and quantitative analysis

Colon sections were fixed in 10% phosphate-buffered formaldehyde (formalin) for 24–48 h, dehydrated, and embedded in paraffin. 5 µm specimens were analyzed by hematoxylin and eosin (H&E, Sigma-Aldrich, St. Louis, MO), trichrome (HT10516, Sigma-Aldrich, St. Louis, MO), or by immunohistochemistry (IHC) using standard protocols. Slides were scanned using a Vectra Automated Quantitative Pathology Imaging System (Perkin Elmer, Waltham, MA). All reported data were verified by a board-certified pathologist, using a standard grading protocol. IHC staining of tissue sections for Ki-67 and CD45 was performed using #27309-I-AP (Ki-67, 1:1000 dilution) and antibodies from Proteintech (Rosemont, IL) and #70257S (CD45, 1:200 dilution, Cell Signaling, Beverly, MA). Expression levels of Ki-67 and CD45 were quantified using inForm software and H-score was calculated as reported previously [28]. Immunofluorescence-IHC using antibodies to Ki-67 (1:100, #27309-I-AP, Proteintech, Rosemont, IL), β-Catenin (1:100, 2698S Cell Signaling, Beverly, MA), ph-S675-β-Catenin (1:70, 4176S Cell Signaling, Beverly, MA), alpha-smooth muscle actin (α-SMA, 1:300, A2547 Sigma-Aldrich, St. Louis, MO), Cldn4 and Cldn7 (1:50 and 1:200, respectively, #16195-I-AP and #10118-I-AP, Proteintech, Rosemont, IL) was used for FFPE tissue sections and organoids embedded in Matrigel using protocols as in [28, 29]. Slides were counterstained with Vectashield mounting medium with DAPI (4′,6-diamidino-2-phenylindole) (H-1200, Vector Labs, Burlingame, CA) to visualize DNA.

Samples were imaged at room temperature (RT) using an SP8 confocal system equipped with an oil-immersion 363 objective with numerical aperture (NA) 1.4 (Leica Microsystems, Buffalo Grove, IL) and LASAF (Leica Application Suite Advanced Fluorescence) software, using NIH ImageJ Imaging Software to quantify area and MetaMorph 7.6.5 software (Molecular Devices, Union City, CA) for measurements of integrated optical density values.

### Luminex assay

The Bio-Plex Pro Cell Signaling Akt Panel 8-Plex Assay kit (#LQ00006JK0K0RR, Bio-Rad Laboratories, Hercules, CA) was used. Tissue samples were lysed using T-PER buffer (ThermoScientific, Waltham, MA), protein concentration established using a BCA Protein Assay Kit (Thermo Scientific, Waltham, MA), and samples incubated overnight in a 96-well plate containing magnetic beads. Subsequently, protein signal was assessed using a Bio-Plex 200 system (Bio-Rad Laboratories, Hercules, CA). Results were processed using GraphPad Prism 6 (GraphPad Software, San Diego, CA) software.

### RNA sequencing and data analysis

Total RNA was isolated from organoids using a Zymo Research Quick-RNA MicroPrep Kit (#R1050) and tested for quality on a Bioanalyzer (Agilent Technologies, Santa Clara, CA). RNA concentrations were determined with a NanoDrop spectrophotometer (Thermo Fisher Scientific, Waltham, MA). Total RNA (standard concentration ≥200 ng/μl, mass ≥10 μg, and RNA integrity number (RIN) ≥ 8.0) was sequenced by Novogene (Sacramento, CA). FastQC was used for read quality assessment (https://www.bioinformatics.babraham.ac.uk/projects/fastqc/). Sequence reads were aligned to the mouse mm10 genome using Tophat2 [30]. The Cufflinks algorithm was used to assemble and quantify transcripts [31], and Cuffdiff to statistically assess expression changes in quantified genes across different conditions [32]. Genes with a false discovery rate of 5% and a fold change of 1.5 were considered differentially expressed. The networks and upstream regulators analysis were generated using Ingenuity Pathway Analysis (IPA) (QIAGEN Inc., https://digitalinsights.qiagen.com/IPA).

### Analysis of publicly available datasets at cBioPortal

The Cancer Genome Atlas (TCGA) PanCancer dataset (594 samples), DFCI (Nature 2016, 619 samples), and Genentech (Nature 2012, 74 samples) datasets for CRC were accessed (July, 2022) and analyzed using tools available at cBioPortal for Cancer Genomics (http://www.cbioportal.org/). In some cases, datasets were merged to increase sample numbers and hence statistical power.

### Statistical analysis

We used Wilcoxon Rank-sum two-tailed tests for pairwise comparisons and 1-way ANOVA for 2 or more group comparisons. Relationships between mutations and patient characteristics were assessed using Fisher exact tests. *P*-values < 0.05 were considered as statistically significant and data presented as mean and S.E.M. Statistical analyses were performed using GraphPad Prism 8 (GraphPad Software, San Diego, CA).

## Results

### Genetic deletion of *Pkd1* reduces CRC tumorigenesis induced by loss of *Apc*

**Fig. 1:**
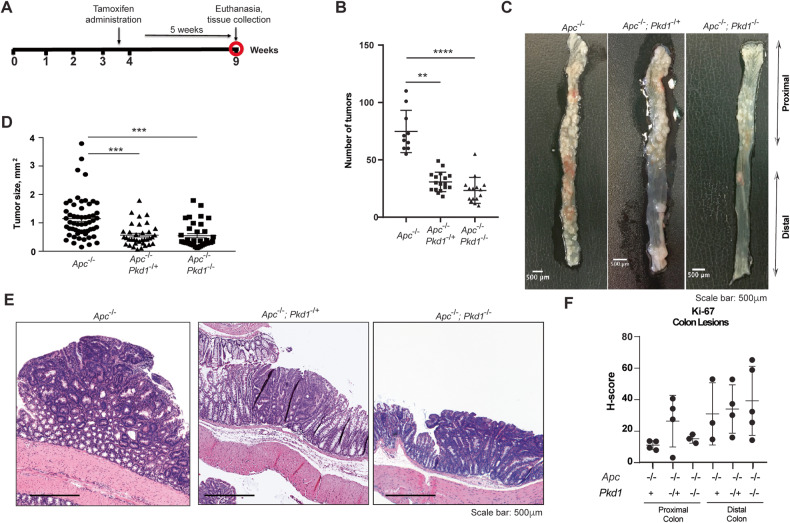
Reduced incidence of CRC in *Apc*^−/−^ mice lacking *Pkd1*. **A** Experimental design. **B** Number of tumors in colon of *Apc*^−/−^ (*n* = 10), *Apc*^−/−^*;Pkd1*^*+/−*^ (*n* = 17*)*, or *Apc*^−/−^*;Pkd1*^−/−^ (*n* = 15) mice. **C** Representative image of colons from mice quantified in (**B**); also see Supp Fig. S1 for additional images. Scale bar, 500 μm. **D** Average size of adenomas at time of euthanasia, in genotypes indicated. **E** Representative H&E stained adenomas or polyps in mice of genotypes indicated. Magnification 4x. Scale bar, 500 μm. **F** Quantification of Ki-67 staining of lesions from genotypes noted, in proximal and distal colon; non-significant differences were observed based on genotype. *p < 0.05; **p < 0.01; ***p < 0.001, in all panels.

To directly assess whether loss of Pkd1 affects the incidence or presentation of colorectal cancer, we used an inducible CRC mouse model in which biallelic loss of a floxed allele of *Apc* (*Apc*^*fl/fl*^) is induced by *CreERT2* (in which tamoxifen induces Cre-lox dependent targeted inactivation), with *CreERT* under the control of the *Cdx2* promoter, and expressed specifically in the epithelium of the distal ileum, cecum, and colon (*Cdx2ERT-Apc*^*fl/fl*^ mice; designated *Apc*^−/−^ hereafter). These mice develop tumors primarily in the distal colon, which is physiologically similar to human CRC [25]. These mice were crossed to mice with a floxed *Pkd1* allele [18–20], to compare tumor formation in *Apc*^−/−^*, Apc*^−/−^*;Pkd1*^*-/+*^, and *Apc*^−/−^*;Pkd1*^−/−^ mice (Fig. 1A, Supp Fig. S1A).

At five weeks after tamoxifen treatment, there was a highly significant reduction in the number of tumors of mice with heterozygous or homozygous loss of *Pkd1* specifically in the same cells as those with loss of *Apc* (e.g., not in the normal colon epithelium). Inactivating *Pkd1* recombination was validated using qRT-PCR probing for Pkd1 exon 1-2 (intact *Pkd1*) and exon 1-5 (deleted exons 2-3-4 in *Pkd1* floxed mice) junctions (see Methods for greater detail). In many cases, reduction of tumor formation was more notable in the distal colon (Fig. 1B, C, Supp Fig. S1B). Quantification of tumor size indicated that tumors emerging in *Apc*^−/−^*;Pkd1*^*-/+*^, and *Apc*^−/−^*;Pkd1*^−/−^ mice were significantly smaller than tumors in *Apc*^−/−^ mice (Fig. 1D, E).

Histopathological assessment by a board-certified pathologist indicated no differences in grade among the distinct tumor genotypes, with all tumors appearing predominantly as adenomas, with rare well-differentiated adenocarcinomas. Ki-67 staining indicated that *Pkd1* genotype did not significantly affect proliferation rate of cells within observed lesions in either the proximal or distal colon (Figs. 1F, S1C). In ADPKD, mutation of *PKD1* is often associated with renal fibrosis, which contributes to scarring and loss of kidney function [33]. Trichrome staining indicated no differences in fibrosis in the CRC lesions of *Apc*^−/−^ and *Apc*^−/−^*;Pkd1*^−/−^ CRC mice, although a trend to higher deposition in the submucosal layers of the colons of *Apc*^−/−^*/ Pkd1*^−/−^ mice (Fig. S2A). We also stained the lesions with alpha-smooth muscle actin (α-SMA) as a marker for a subset of activated myofibroblasts, which are important effectors of tissue fibrogenesis (Fig. S2B). Interestingly, α-SMA expression was expressed in the muscularis layer of the colons of *Apc*^−/−^*/Pkd1*^−/−^ mice versus *Apc*^−/−^ mice, and to a greater extent in the *Apc*^−/−^*/Pkd1*^−/−^ lesions, paralleling results in ADPKD.

### Reduced CRC incidence in *Pkd1*-deficient colons is partially mimicked in organoids

**Fig. 2:**
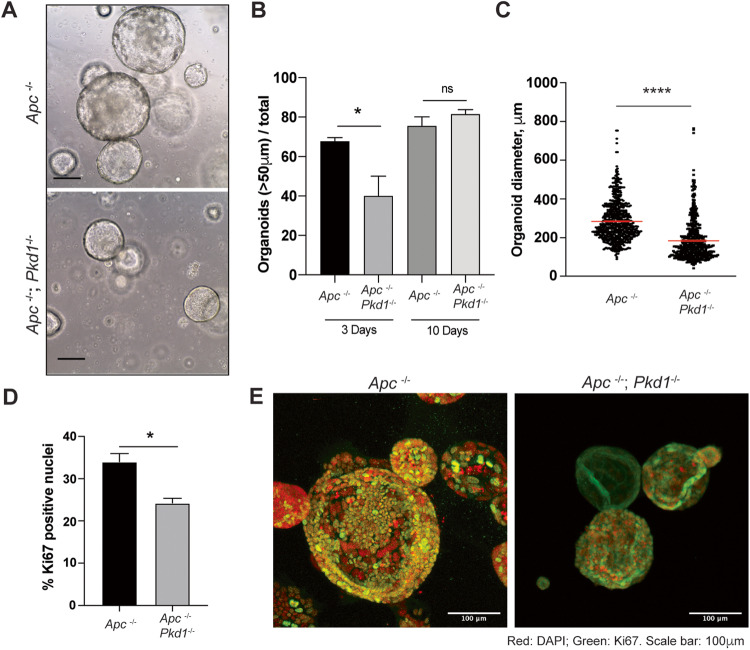
Reduced growth of CRC-derived *Apc*^−/−^ organoids lacking *Pkd1*. **A** Representative images of organoid growth in Matrigel. Scale bar, 50 μm. **B** Seeding efficiency of organoids based on genotype, based on a size threshold of 50 μm. **C** Average size of organoids from *Apc*^−/−^ versus *Apc*^−/−^*;Pkd1*^−/−^ genotypes after 7 days.^.^ **D**, **E** Quantification (**D**) and representative images (**E**) of Ki-67 staining of organoids of genotypes indicated. Scale bar, 100 μm. ns, not significant; *p < 0.05; **p < 0.01; ***p < 0.001, ****p < 0.0001 in all panels.

The reduced incidence of *Pkd1*-deficient tumors might solely reflect cell intrinsic consequences of *Pkd1* loss in colon epithelial cells or might instead reflect the altered interaction of PKD1-deficient epithelial cells with the nascent tumor microenvironment. To address these possibilities, we treated *Apc*^−/−^ and *Apc*^−/−^*Pkd1*^−/−^ mice with tamoxifen, and after 5 weeks collected visible lesions and generated organoids in Matrigel (Fig. 2). Gross morphology of organoids and efficiency of seeded cells in forming organoids were not affected by *Pkd1* genotype, but there was a delay in organoid formation with *Apc*^−/−^*Pkd1*^−/−^ relative to *Apc*^−/−^ cells (Fig. 2A, B), reflecting reduced growth rate. Overall, after 7 days in culture, *Apc*^−/−^*Pkd1*^−/−^ organoids were significantly smaller than *Apc*^−/−^ organoids on average (mean diameter 284.9 versus 184.7 microns; *p* < 0.0001) (Fig. 2C). In contrast to in vivo observations, Ki-67 staining of colonies indicated a somewhat reduced number of proliferating cells among those with the *Apc*^−/−^*Pkd1*^−/−^ genotype after 10-14 days in culture (Fig. 2D, E). This difference may reflect the higher overall proliferative index occuring in vitro versus in vivo, or alternatively, some compensation in vivo for the antiproliferative effect of Pkd1 loss.

### Genetic deletion of *Pkd1* elevates Wnt/Ctnnb1-dependent transcription

**Fig. 3:**
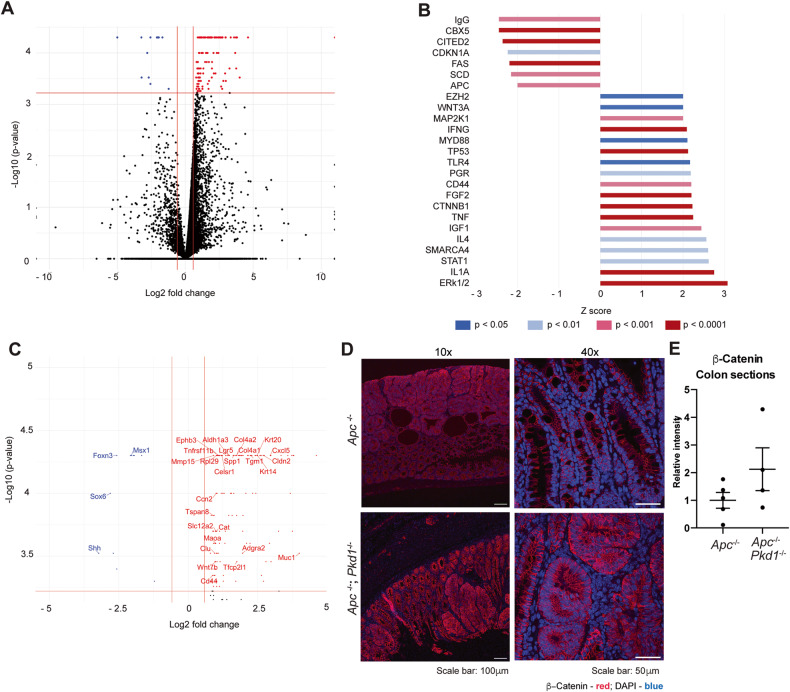
RNA sequencing of organoids indicates loss of *Pkd1* elevates Wnt/Ctnnb1 signaling. **A** Volcano plot showing distribution of genes differentially expressed in organoids of the *Apc*^−/−^*;Pkd1*^−/−^ versus *Apc*^−/−^ genotypes, based on statistical significance (Y axis, *p* value) and log2 fold change (x axis). **B** Waterfall plot demonstrating upstream transcriptional regulators statistically significantly changed in *Apc*^−/−^*;Pkd1*^−/−^ versus *Apc*^−/−^ organoids. **C** Expanded volcano plot focused on genes above cutoffs for statistical significance and magnitude of change, from A. Gene names shown reflect those linked to CTNNB1 signaling as targets identified by Ingenuity (23 of 30 total genes above cut-off values) or as regulators, based on literature (in italics). **D**, **E** Immunofluorescence staining of lesions from *Apc*^−/−^*;Pkd1*^−/−^ versus *Apc*^−/−^ mice colons for β-catenin (**D**), at 10x (left images) and 40x (right images), with quantification of signal intensity (**E**).

To gain insight into signaling differences induced by loss of *Pkd1*, performed RNAseq analysis on organoids prepared from the *Apc*^−/−^ and *Apc*^−/−^*Pkd1*^−/−^ genotypes to gain a more detailed insight into the transcriptional consequences of loss of *Pkd1* specifically in *Apc*^−/−^ colorectal epithelia. Loss of *Pkd1* significantly increased the expression of 127 genes, and decreased the expression of 11 genes, based on a threshold of 1.5-fold-change and a *p*-value < 0.05 to establish significance (Fig. 3A, Supp Table S1). Surprisingly, Ingenuity pathway analysis (Fig. 3B) identified a complex set of changes, including upregulation of multiple pro-proliferative pathways, including WNT/CTNNB1, ERK1, and IGF1, as well as proinflammatory signaling pathways associated with IFNG and IL1A. However, antiproliferative pathways were also upregulated, including numerous transcripts regulated by TP53, indicating opposing signals. In addition, Luminex analysis of a panel of signaling proteins involved in mediating cell growth revealed no significant difference between the *Apc*^−/−^ and *Apc*^−/−^*Pkd1*^−/−^ genotypes (Supp Fig. S2C).

Given the activation of the CTNNB1 pathway is a primary proliferation-promoting consequence of APC loss in colon crypts [34], and given the large number of upregulated transcripts associated with CTTNB1 (Fig. 3C), we further probed activity of this pathway. immunohistochemical (IHC) analysis of pre-malignant colon tissue, polyps, and adenomas from *Apc*^−/−^*Pkd1*^−/−^ versus *Apc*^−/−^ mice confirmed elevated CTNNB1 expression and active (S^675^-phosphorylated) CTNNB1 associated with the *Apc*^−/−^*Pkd1*^−/−^ genotype, as well as in *Apc*^−/−^*Pkd1*^−/−^ organoids (Fig. 3D, E, Supp Fig. S3A, B). In all cases, although there was not a marked shift of CTNNB1 from the periphery to the nucleus, the elevation of S^675^-phosphorylated CTNNB1 [35], was compatible with greater pathway activity and upregulation of CTNNB1-dependent transcripts.

### Loss of *Pkd1* increases colon barrier function

**Fig. 4:**
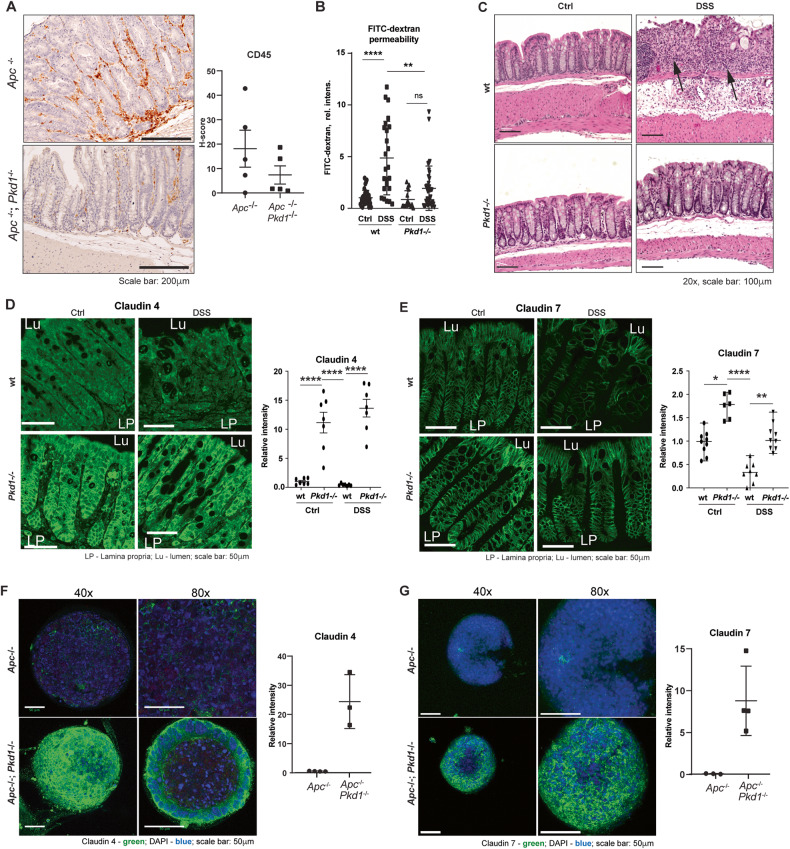
Pkd1 regulation of epithelial barrier function and claudin expression. **A** Immunohistochemical staining of CD45 expression in colon lesions of *Apc*^−/−^ versus *Apc*^−/−^*;Pkd1*^−/−^ mice. Left—representative images, scale bars, 200 μm. Right—quantification. **B** Quantification of FITC-dextran levels in serum of dextran sodium sulfate (DSS)- or control water (Ctrl)-treated mice. **C** Representative H&E images of colon tissue from *Pkd1*^*wt*^ versus *Pkd1*^−/−^ mice treated with water or DSS. Scale bars, 100 μm. Measurements of mouse weight during treatment are located in Fig S4B. **D**, **E** Representative images (left) and quantification (right) of expression of CLDN4 (**D**) and CLDN7 (**E**) in *wt* versus *Pkd1*^−/−^ colon epithelia. Scale bars 50 μm. **F**, **G** Representative images (left) and quantification (right) of CLDN4 and CLDN7 staining in organoids. Blue, DAPI; green, claudins. Scale bars 50 μm. *p < 0.05; **p < 0.01; ***p < 0.001; ****p < 0.0001 for all graphs.

Intriguingly, loss of PKD1 function in ADPKD enables the epithelial monolayer to increase the transit of fluids into the cyst and to form a non-leaky barrier that can withstand high hydrostatic pressure within the cyst [36, 37]. Conversely, CRC risk is elevated by factors that reduce intestinal barrier function [38–41], which promotes increased inflammation in human patients and mouse models [23, 42]. In this process, designated Tumor-Elicited Inflammation (TEI), colonies of microbes adjacent to pre-malignant colon tissue gradually infiltrate the epithelial layer. Interaction of microbes with recruited myeloid cells and tumor cells elicits IL-23/IL-17 signaling that promotes tumor growth and initiates a feed-forward cycle, reprogramming of tight junctions to further reduce epithelial barrier permeability [23, 42, 43]. This was suggestive, because comparison of *Apc*^−/−^*Pkd1*^−/−^ versus *Apc*^−/−^ tumors identified a trend towards lower CD45-positive leukocyte infiltration in the *Apc*^−/−^*Pkd1*^−/−^ genotype (Fig. 4A).

To test the hypothesis that loss of *Pkd1* influenced gut barrier function, positioning it to reduce TEI, we used treatment with dextran-sodium sulfate (DSS), an irritant that damages the gut epithelium, eliminating the protective mucin lining of the gut epithelium, and causing epithelial cell loss and tissue damage; reduction in epithelial barrier function typically sensitizes mice to DSS [44, 45]. We treated 15-week-old *Pkd1*^*+/+*^ (designated wild-type [*wt*] hereafter) or *Pkd1*^−/−^ mice (with loss of *Pkd1* induced in all tissues by tamoxifen injection at days P35 and P36) for 5 days with 2.5% DSS in drinking water, versus water-only controls. We then gave FITC-dextran tracer by oral gavage and bled mice after 3 h to collect serum and analyzed the appearance of FITC-dextran in the serum, as in [23] (Fig. 4B). Whereas DSS elevated serum levels 5-fold over those in untreated *wt* mice, *Pkd1*^−/−^ mice showed only a 2-fold, statistically insignificant increase in FITC-dextran in serum, strongly suggesting reinforced barrier function. Further, immunohistochemical analysis of colonic tissue from vehicle- or DSS-treated *wt* and *Pkd1*^−/−^ mice shows that PKD1 deficiency substantially reduces the tissue damage induced by response to DSS (Fig. 4C). In wt mice, DSS induced a complete loss of the organized mucosal layer, including loss of mucus-producing cells, irregularity and expansion of the submucosal layer, and evidence of infiltration of immune cells (arrows, Fig. 4C). In contrast, while some defects in the mucosal layer were observed in DSS-treated *Pkd1*^−/−^ mice, they were much less severe in all mice. In addition, while DSS promoted the infiltration of CD45+ leukocytes, infiltration was limited to submucosal layer in DSS-treated animals lacking *Pkd1*, whereas much more pervasive leukocyte infiltration characterized DSS-treated mice with wt *Pkd1* (Fig. S4A).

### Loss of *Pkd1* in the colon induces claudins that promote impermeable barriers

In developing a hypothesis for Pkd1 action, we considered the fact that mechanistically, *Pkd1* loss in ADPKD produces cysts in part by upregulating claudins 4 and 7 (CLDN4, CLDN7) and others that similarly induce impermeable tight junctions, while downregulating those that permit barrier permeability [46, 47]. In human CRC, an early step in TEI is the post-transcriptional downregulation of CLDN7 and CLDN4 [23], which contributes to the penetration of bacteria and their inflammatory products [23]; loss of CLDN7 has been shown to promote colorectal inflammation and tumorigenesis [48]. Comparison of colon tissue from *wt* and *Pkd1*^−/−^ mice indicated significantly elevated levels of CLDN4 and CLDN7 in *Pkd1*^−/−^ colonic epithelia (Fig. 4D, E). Similar elevation of CLDN4 and CLDN7 expression was observed in *Apc*^−/−^*Pkd1*^−/−^ versus *Apc*^−/−^ organoids (Fig. 4F, G). Together with results from DSS administration, these data suggest loss of *Pkd1* induces claudins to decrease permeability of the colon epithelium, restricting inflammation- and proliferation-promoting TEI.

### Loss of *Pkd1* in colon epithelial cells influences response to targeted and cytotoxic therapies

**Fig. 5:**
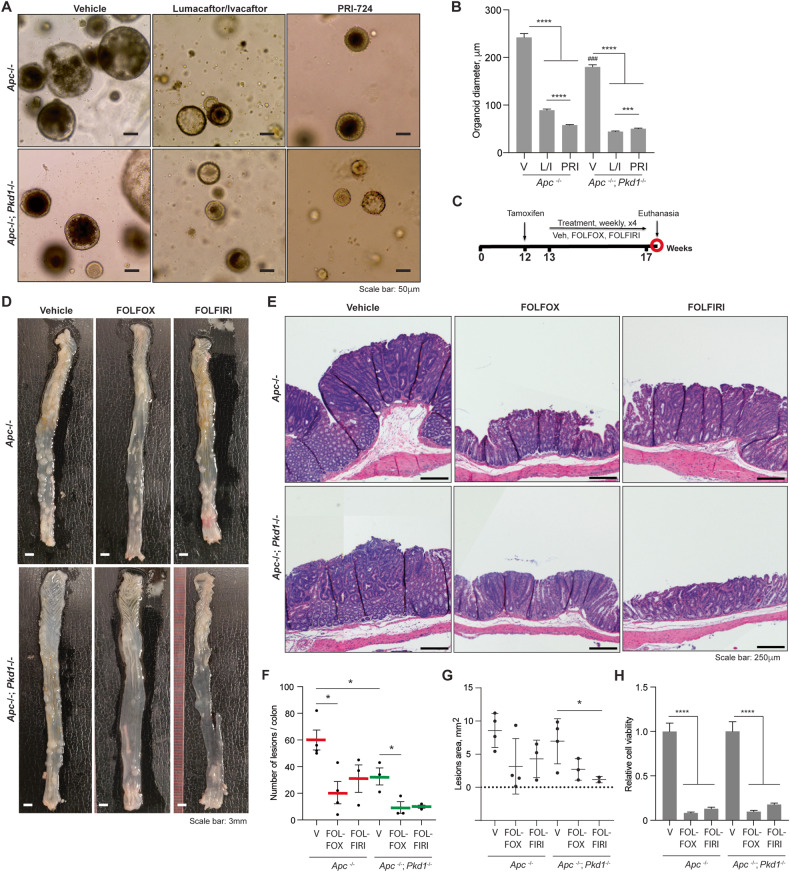
Response to drugs targeting CFTR and CTNNB1, and chemotherapy, based on *Pkd1* genotype. **A**, **B** Representative images (**A**) and quantitation (**B**) of organoid size following treatment with vehicle, the CFTR activator pair lumacaftor/ivacaftor (L/I), or the CTNNB1 inhibitor PRI-724 (PRI), based on *Apc*^−/−^ versus *Apc*^−/−^*;Pkd1*^−/−^ genotype. Scale bars on (**A**), 50 μm. **C** Experimental design to assess in vivo response to FOLFIRI or FOLFOX. **D**, **E** Representative image of colons from mice treated with FOLFOX and FOLFIRI; gross presentation (**D**) and H&E-stained tissue (**E**). **F**, **G** Quantification of number of lesions based on gross assessment of number in colon (**F**) and area, based on histopathological assessment (**G**) following treatment with vehicle, FOLFIRI, or FOLFOX, in genotypes indicated. **H** Quantitation of viability based on CellTiterGlo of organoid cultures following treatment with vehicle, FOLFIRI, or FOLFOX, in indicated genotypes, with data normalized to vehicle-treated *Apc*^−/−^ organoids. *p < 0.05; **p < 0.01; ***p < 0.001; ****p < 0.0001 for all graphs; in (**B**), ^###^*p* < 0.001; compared to *Apc*^−/−^ vehicle.

To gain insight into the mechanism by which *Pkd1* loss reduced tumor growth, we considered that in ADPKD, *Pkd1* loss activates the cystic fibrosis transmembrane receptor (CFTR), contributing to polarized ion secretion [49]. Notably, CFTR has been reported to act as a tumor suppressor in CRC [50], with cystic fibrosis patients prone to elevated CRC risk [51], and loss of CFTR previously shown to reduce expression of CLDN7 [52]. To gain insight into the role of CFTR signaling in PKD1 activity in the colon, we compared the growth of *Apc*^−/−^*Pkd1*^−/−^ versus *Apc*^−/−^ organoids treated with a CFTR-activating drug combination lumacaftor/ivacaftor (L/I) [53]. We benchmarked these results to the effect of *Apc*^−/−^*Pkd1*^−/−^ versus *Apc*^−/−^ organoids treated with the WNT pathway inhibitor PRI-724 [54], which inhibits the interaction between CTNNB1 and its transcriptional coactivator CBP, and should be active in all CRC arising from an *Apc*^−/−^ genotype. Both drugs were very effective in reducing organoid growth, indicating the importance of both WNT activity and CFTR inhibition for allowing colon cell growth (Fig. 5A, B). Interestingly, while *Apc*^−/−^ organoids responded more strongly to inhibition of CTNNB1 than activation of CFTR, *Apc*^−/−^*Pkd1*^−/−^ organoids responded comparably to both drugs, suggesting greater importance of CFTR activation in regulating growth of organoids lacking *Pkd1*.

Standard of care treatment for CRC typically involves used of DNA-damaging cytotoxic chemotherapies, FOLFIRI and FOLFOX [55]. To determine if Pkd1 loss affected response to these drugs, we induced loss of *Apc* and *Pkd1* with tamoxifen treatment of 12-week-old mice, and commencing at week 13, treated mice weekly with either vehicle, or with standard DNA-damaging cytotoxic chemotherapies for CRC, FOLFIRI and FOLFOX (Fig. 5C). Both treatments were effective at eliminating >60% of lesions relative to vehicle in both *Apc*^−/−^ and >*Apc*^−/−^*Pkd1*^−/−^ mice (Fig. 5D–G). Given the lower number and size of lesions occurring in *Apc*^−/−^*Pkd1*^−/−^ mice, almost no adenomas were observed in these animals following drug treatments. In contrast to results with targeted inhibitors, treatment of organoids with FOLFIRI or FOLFOX was similarly effective in reducing organoid growth in both genotypes (Fig. 5H).

### *PKD1* mutation and expression profiles in human CRC

**Fig. 6:**
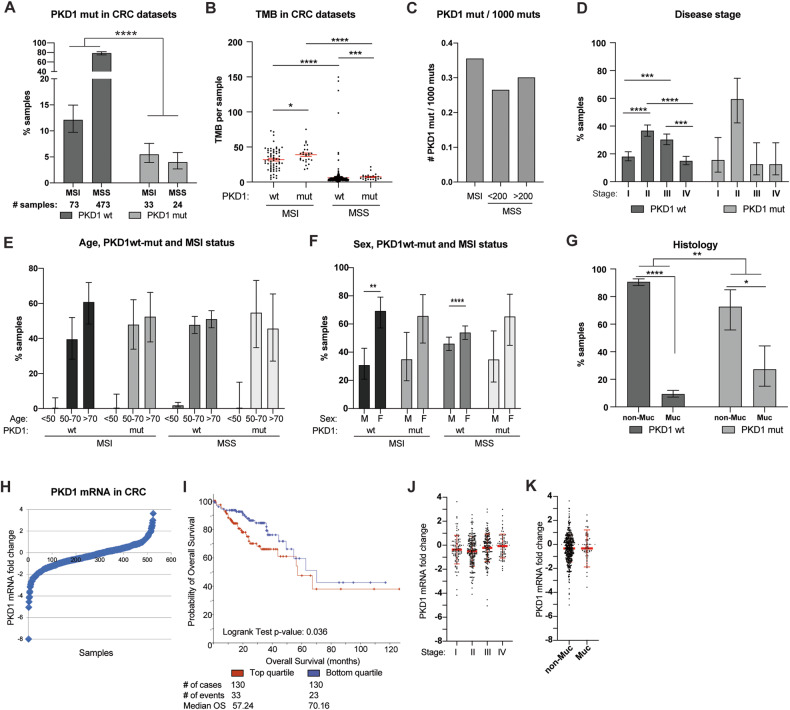
Analysis of functional impact of *PKD1* mutations in human CRC datasets available at cBioPortal. **A** Proportion of *PKD1* wild type (wt) versus mutated (mut) human CRC specimens, separately analyze based on microsatellite stability status (MSI vs. MSS). **B** Association of *PKD1* mutational status with TMB in MSI and MSS samples. **C** Distribution of *PKD1* mutations normalized by 1000 mutations per sample. **D** Relationship of *PKD1* mutation status and disease stage at diagnosis. **E** Relationship of PKD1 mutations versus age of patient at diagnosis. **F** Segregation of *PKD1* mutations by sex in MSI versus MSS tumors. **G** Relationship between *PKD1* mutation and tumor histology in CRCs. *p < 0.05; **p < 0.01; ***p < 0.001; ****p < 0.0001 for all graphs. **H** Range of PKD1 mRNA expression in 524 CRC samples reported in cBioPortal. **I** Overall survival following initial treatment of CRC tumors expressing top quartile (red) or bottom quartile (blue) *PKD1* mRNA expression. Note, datasets with survival information do not include MSS/MSI status. **J** Distribution of mRNA expression levels in CRC tumors analyzed at stages I–IV. **K** Expression of PKD1 mRNA in tumors of mucinous versus non-mucinous histology.

We analyzed the profile of *PKD1* mutations in CRC datasets reported in cBioPortal, to determine if *PKD1*-mutated tumors had any specific distinguishing features (Fig. 6). In the 603 CRC samples for which data was available, 497 tumors were microsatellite stable (MSS), and 106 had microsatellite instability (MSI) (82.4 versus 17.6%) (Fig. 6A). Among the 546 *PKD1 wt* tumors, 473 (87%) were MSS; in contrast, among the 57 tumors with non-synonymous mutations in *PKD1* and with annotation as MSS or MSI, only 24 were MSS (42%), indicating a significant bias to occurrence in MSI. Typically, for both the MSI and MSS cohorts, tumors with *PKD1* mutations were associated with higher TMB than tumors with wild-type (wt) *PKD1* (Fig. 6B, C). Based on rules for determining degree of damaging effect established through studies of ADPKD-associated mutations [10], approximately 1/3 of the *PKD1* mutations were predicting to be non-damaging, which may suggest a passenger role. However, overall, *PKD1*-mutated tumors tended to be observed at earlier stages than *PKD1* tumors (although the observation did not reach statistical significance due the limited number of *PKD1*-mutated tumors) (Fig. 6D). The age range of diagnosis for *PKD1*-mutated tumors was similar to that of *PKD1* wt tumors (Fig. 6E), as was the distribution by sex (Fig. 6F). We also examined distribution of *PKD1* mutations among tumors of distinct histology, including mucinous versus non-mucinous (Fig. 6G). Although non-mucinous tumors predominated in both cohorts, a significantly higher percentage *PKD1*-mutated tumors were likely to be mucinous than were *PKD1* wild-type tumors.

*PKD1* mRNA expression varied over a significant range (~12-fold) in human CRCs (Fig. 6H). Notably, comparison of CRC with PKD1 expression in the highest quartile versus the lowest quartile showed significantly lower overall survival after treatment in individuals with tumors expressing the higher levels of *PKD1* (Fig. 6I). Analysis of PKD1 expression by tumor stage indicated no significant difference in *PKD1* expression at distinct stages of CRC (Fig. 6J), suggesting the survival differences were not attributable to higher *PKD1* expression in later stage tumors. Given the differences seen in the frequency of *PKD1* mutations in mucinous versus non-mucinous tumors, we also investigated mRNA expression differences for *PKD1* in these histologies, but noted no differences (Fig. 6K).

## Discussion

As major conclusions, this study for the first time directly demonstrates that an intact *Pkd1* gene supports early stages of CRC formation, and that loss of PKD1 is tumor suppressive in adenoma formation mediated by loss of APC in a mouse model. These tumor suppressive activities of PKD1 loss are not linked to suppression of pro-proliferative signaling by CTNNB1 or other oncogenic pathways, but associated with increased colon barrier function, increased expression of claudins that mediate increased colon epithelial barrier function, and decreased leukocyte infiltration. Although loss of PKD1 does not differentially affect the response of organoids or tumors to cytotoxic regimens, the lower baseline of tumors in *Apc*^−/−^*Pkd1*^−/−^ mice results in a more effective reduction in total tumor burden following treatment with these agents. Finally, analysis of public human data suggest *PKD1* mutations are more likely to occur in MSI versus MSS CRC, and to be found in mucinous versus non-mucinous tumors, differing from the distribution of *PKD*1 *wt* CRC; further, expression of higher levels of *PKD1* is associated with poorer outcomes for CRC recurrence and survival.

Extensive studies of functional changes in cell and tissue organization in the context of ADPKD have demonstrated an extremely pleiotropic activity, in which mutations reducing PKD1 expression or function produce phenotypes similar to those observed in tumors and described as the “hallmarks of cancer”, albeit typically in attenuated form [56]. However, in spite of these many similarities in phenotypes and signaling, ADPKD cysts are absolutely distinguished from tumors in their retention of a monolayer growth habit. This distinction argues for a strong PKD1-dependent inhibitory signal restricting invasive growth. Stabilization of impermeable tight junctions (TJs)—an essential feature of cyst pathogenesis mediated by claudin reprogramming [47]—is a strong candidate for such an inhibitory signal, as loss of TJs is the first step in the process of EMT that triggers invasion. Specifically, our data is compatible with the idea that loss of *Pkd1* activates a CFTR-CLDN7 signaling axis to restrict TEI, based on data in this study and independent publications showing enhanced intestinal permeability and colon inflammation following inactivation of *Cftr* and *Cldn7* [46, 48, 52, 57, 58]. In future work, it would be of interest to compare the activity of Pkd1 mutation in restricting CRC formation in azoxymethane/DSS-induced tumorigenesis models [59] to the effect observed here in models based on mutation of *Apc*.

There are numerous differences between the biology of ADPKD and CRC. In one notable example, in normal kidney tissues and in ADPKD, PKD1 function is typically linked to the action of the protein on cilia [60, 61]; in contrast, few if any colon epithelial cells are ciliated [62, 63]. In ADPKD, signaling pathways activated in renal cysts by loss of *PKD1* include many known to be activated and growth-promoting in cancer including WNT/CTNNB1, PI3K/AKT, mTOR/S6K, RAF/MEK/ERK, SRC, MYC, AURKA, and others [56, 64, 65]. Some prior studies of PKD1 have documented a role of overexpressed PKD1 in activating of some of these signaling pathways in CRC and other cancer cells [16, 66]. Other pathways influenced by loss of PKD1 in ADPKD include planar cell polarity (non-canonical WNT) and the Hippo contact-inhibition associated pathways [67, 68]; these also are relevant to CRC etiology (e.g., [69]).

This study does not assign a single specific signaling mechanism by which loss of PKD1 inhibits CRC, although it excludes inhibition of WNT/CTNNB1 as one possibility, based on elevation of this pathway in *Pkd1*-mutated murine adenomas or in human tumors bearing somatic *PKD1* mutations. A curious element of this study is the fact that CTNNB1 total levels are elevated, and WNT/CTNNB1-dependent transcript levels are elevated, but we are unable to detect a shift in distribution from cell periphery to nucleus for the CTNNB1 protein. This suggests that the increase in CTNNB1-dependent transcription reflects the overall increase in the levels of cellular CTNNB1, but not a specific activation of the pathway. In contrast, there is little evidence for *PKD1* mutations stimulating activity of growth-inhibitory signaling proteins, with the exception of CFTR. A point bearing further study is the relationship of Pkd1 mutation and reduction of proliferation (Ki-67 staining), which is striking in organoids, but less notable in vivo in this study. One explanation might be the fact that while loss of Pkd1 is growth-inhibitory in a cell-autonomous manner in vitro, factors in the nascent tumor microenvironment—perhaps mediated by immune system components—ameliorate this effect in vivo. In this context, it is intriguing that trichrome and α-SMA staining of lesions indicates altered collagen deposition patterns and increased numbers of myofibroblasts associated with the *Apc*^−/−^*Pkd1*^−/−^ genotype. Myofibroblasts and their secreted extracellular matrix can have significant effects on tumor growth, immunological response, and response to treatment [70]. The fact that leukocyte infiltration patterns are altered and reduced by absence of Pkd1, both in lesions and following treatment with DSS, may in part be influenced indirectly by changes in the tumor microenvironment—a topic of considerable interest for future investigations.

Finally, earlier studies have connected *PKD1* mutations and CRC risk [8], and also suggested that elevated levels of PKD1 in late-stage tumors and tumor-derived cell lines are associated with more invasive phenotypes due to increased EMT and migration, and worse survival [16]. By reinforcing and extending these earlier studies, this study raises the possibility that not only the risk, but also the presentation and clinical response of CRC—and potentially inflammatory bowel disease—may differ in individuals with ADPKD versus the general population. The tendency of *PKD1* mutations to occur in MSI tumors identified here is of interest. Given the distinct biology of MSS tumors, which typically (~80%) originate with APC mutations, and MSI tumors, where APC mutations are less common and other activating mutations are more prevalent [71], it is possible that *PKD1* mutation and restriction of TEI is selectively detrimental in the MSS sequence of tumor formation and progression; although additional work is required to determine whether the relationship is causal or correlative. Estimates of the rate of APDKD in the population suggest as many as 1 in 1000 individuals may have inherited gene variants damaging *PKD1* or its partner, *PKD2* [12, 72]; reflecting ~300,000 individuals in the United States. Data from this study suggests that studies examining the features of diseases affecting the gastrointestinal tract in the ADPKD population would be merited.

### Supplementary information

Supplemental Figures, Tables, Legends

## Data Availability

RNA sequencing data have been deposited in the NCBI database (url pending). Other primary data are available via application to the corresponding author.

## References

[1] American_Cancer_Society. Colorectal cancer facts and figures 2014–2016. Atlanta: American Cancer Society I; 2014.

[2] Fearon ER, Vogelstein B (1990). A genetic model for colorectal tumorigenesis. Cell.

[3] Jones S, Chen WD, Parmigiani G, Diehl F, Beerenwinkel N, Antal T (2008). Comparative lesion sequencing provides insights into tumor evolution. Proc Natl Acad Sci USA.

[4] Glaire MA, Brown M, Church DN, Tomlinson I (2017). Cancer predisposition syndromes: lessons for truly precision medicine. J Pathol.

[5] Scarpa A, Chang DK, Nones K, Corbo V, Patch AM, Bailey P (2017). Whole-genome landscape of pancreatic neuroendocrine tumours. Nature.

[6] Ma H, Brosens LAA, Offerhaus GJA, Giardiello FM, de Leng WWJ, Montgomery EA (2018). Pathology and genetics of hereditary colorectal cancer. Pathology.

[7] Huyghe JR, Bien SA, Harrison TA, Kang HM, Chen S, Schmit SL (2019). Discovery of common and rare genetic risk variants for colorectal cancer. Nat Genet.

[8] Wetmore JB, Calvet JP, Yu AS, Lynch CF, Wang CJ, Kasiske BL (2014). Polycystic kidney disease and cancer after renal transplantation. J Am Soc Nephrol.

[9] Cornec-Le Gall E, Torres VE, Harris PC (2018). Genetic complexity of autosomal dominant polycystic kidney and liver diseases. J Am Soc Nephrol.

[10] Heyer CM, Sundsbak JL, Abebe KZ, Chapman AB, Torres VE, Grantham JJ (2016). Predicted mutation strength of nontruncating PKD1 mutations aids genotype-phenotype correlations in autosomal dominant polycystic kidney disease. J Am Soc Nephrol.

[11] Ward CJ, Wu Y, Johnson RA, Woollard JR, Bergstralh EJ, Cicek MS (2011). Germline PKHD1 mutations are protective against colorectal cancer. Hum Genet.

[12] Cornec-Le Gall E, Alam A, Perrone RD (2019). Autosomal dominant polycystic kidney disease. Lancet.

[13] Geng L, Segal Y, Pavlova A, Barros EJ, Lohning C, Lu W (1997). Distribution and developmentally regulated expression of murine polycystin. Am J Physiol.

[14] Conduit SE, Hakim S, Feeney SJ, Ooms LM, Dyson JM, Abud HE (2019). Beta-catenin ablation exacerbates polycystic kidney disease progression. Hum Mol Genet.

[15] Wuebken A, Schmidt-Ott KM (2011). WNT/beta-catenin signaling in polycystic kidney disease. Kidney Int.

[16] Gargalionis AN, Korkolopoulou P, Farmaki E, Piperi C, Dalagiorgou G, Adamopoulos C (2015). Polycystin-1 and polycystin-2 are involved in the acquisition of aggressive phenotypes in colorectal cancer. Int J Cancer.

[17] Outeda P, Huso DL, Fisher SA, Halushka MK, Kim H, Qian F (2014). Polycystin signaling is required for directed endothelial cell migration and lymphatic development. Cell reports.

[18] Piontek K, Menezes LF, Garcia-Gonzalez MA, Huso DL, Germino GG (2007). A critical developmental switch defines the kinetics of kidney cyst formation after loss of Pkd1. Nat Med.

[19] Piontek KB, Huso DL, Grinberg A, Liu L, Bedja D, Zhao H (2004). A functional floxed allele of Pkd1 that can be conditionally inactivated in vivo. J Am Soc Nephrol.

[20] Nikonova AS, Plotnikova OV, Serzhanova V, Efimov A, Bogush I, Cai KQ (2014). Nedd9 restrains renal cystogenesis in Pkd1-/- mice. Proc Natl Acad Sci USA.

[21] Nikonova AS, Deneka AY, Kiseleva AA, Korobeynikov V, Gaponova A, Serebriiskii IG (2018). Ganetespib limits ciliation and cystogenesis in autosomal-dominant polycystic kidney disease (ADPKD). FASEB J.

[22] Seeger-Nukpezah T, Proia DA, Egleston BL, Nikonova AS, Kent T, Cai KQ (2013). Inhibiting the HSP90 chaperone slows cyst growth in a mouse model of autosomal dominant polycystic kidney disease. Proc Natl Acad Sci USA.

[23] Grivennikov SI, Wang K, Mucida D, Stewart CA, Schnabl B, Jauch D (2012). Adenoma-linked barrier defects and microbial products drive IL-23/IL-17-mediated tumour growth. Nature.

[24] Hinoi T, Loda M, Fearon ER (2003). Silencing of CDX2 expression in colon cancer via a dominant repression pathway. J Biol Chem.

[25] Hinoi T, Akyol A, Theisen BK, Ferguson DO, Greenson JK, Williams BO (2007). Mouse model of colonic adenoma-carcinoma progression based on somatic Apc inactivation. Cancer Res.

[26] Billips LG, Petitte D, Landreth KS (1990). Bone marrow stromal cell regulation of B lymphopoiesis: interleukin-1 (IL-1) and IL-4 regulate stromal cell support of pre-B cell production in vitro. Blood.

[27] Boj SF, Hwang CI, Baker LA, Chio II, Engle DD, Corbo V (2015). Organoid models of human and mouse ductal pancreatic cancer. Cell.

[28] Deneka AY, Kopp MC, Nikonova AS, Gaponova AV, Kiseleva AA, Hensley HH (2021). Nedd9 restrains autophagy to limit growth of early stage non-small cell lung cancer. Cancer Res.

[29] Debnath J, Muthuswamy SK, Brugge JS (2003). Morphogenesis and oncogenesis of MCF-10A mammary epithelial acini grown in three-dimensional basement membrane cultures. Methods.

[30] Trapnell C, Pachter L, Salzberg SL (2009). TopHat: discovering splice junctions with RNA-Seq. Bioinformatics.

[31] Trapnell C, Williams BA, Pertea G, Mortazavi A, Kwan G, van Baren MJ (2010). Transcript assembly and quantification by RNA-Seq reveals unannotated transcripts and isoform switching during cell differentiation. Nat Biotechnol.

[32] Trapnell C, Hendrickson DG, Sauvageau M, Goff L, Rinn JL, Pachter L (2013). Differential analysis of gene regulation at transcript resolution with RNA-seq. Nat Biotechnol.

[33] Fragiadaki M, Macleod FM, Ong ACM (2020). The controversial role of fibrosis in autosomal dominant polycystic kidney disease. Int J Mol Sci.

[34] van de Wetering M, Sancho E, Verweij C, de Lau W, Oving I, Hurlstone A (2002). The beta-catenin/TCF-4 complex imposes a crypt progenitor phenotype on colorectal cancer cells. Cell.

[35] Zhu G, Wang Y, Huang B, Liang J, Ding Y, Xu A (2012). A Rac1/PAK1 cascade controls beta-catenin activation in colon cancer cells. Oncogene.

[36] Grantham JJ (1990). Polycystic kidney disease: neoplasia in disguise. Am J Kidney Dis.

[37] Grantham JJ, Nair V, Winklhoffer F. Cystic diseases of the kidney. In: Brenner BM, editors. Brenner & rector’s the kidney. Philadelphia: WB Saunders Company; 2000. p. 1699–730.

[38] Choi CR, Bakir IA, Hart AL, Graham TA (2017). Clonal evolution of colorectal cancer in IBD. Nat Rev Gastroenterol Hepatol.

[39] Gonzalez N, Prieto I, Del Puerto-Nevado L, Portal-Nunez S, Ardura JA, Corton M (2017). 2017 update on the relationship between diabetes and colorectal cancer: epidemiology, potential molecular mechanisms and therapeutic implications. Oncotarget.

[40] Park J, Morley TS, Kim M, Clegg DJ, Scherer PE (2014). Obesity and cancer-mechanisms underlying tumour progression and recurrence. Nat Rev Endocrinol.

[41] Rasool S, Kadla SA, Rasool V, Ganai BA (2013). A comparative overview of general risk factors associated with the incidence of colorectal cancer. Tumour biology : the journal of the International Society for Oncodevelopmental Biology and Medicine.

[42] Wang K, Kim MK, Di Caro G, Wong J, Shalapour S, Wan J (2014). Interleukin-17 receptor a signaling in transformed enterocytes promotes early colorectal tumorigenesis. Immunity.

[43] Taniguchi K, Wu LW, Grivennikov SI, de Jong PR, Lian I, Yu FX (2015). A gp130-Src-YAP module links inflammation to epithelial regeneration. Nature.

[44] Meira LB, Bugni JM, Green SL, Lee CW, Pang B, Borenshtein D (2008). DNA damage induced by chronic inflammation contributes to colon carcinogenesis in mice. J Clin Invest.

[45] Suzuki R, Kohno H, Sugie S, Nakagama H, Tanaka T (2006). Strain differences in the susceptibility to azoxymethane and dextran sodium sulfate-induced colon carcinogenesis in mice. Carcinogenesis.

[46] Gunzel D, Yu AS (2013). Claudins and the modulation of tight junction permeability. Physiol Rev.

[47] Yu AS, Kanzawa SA, Usorov A, Lantinga-van Leeuwen IS, Peters DJ (2008). Tight junction composition is altered in the epithelium of polycystic kidneys. J Pathol.

[48] Tanaka H, Takechi M, Kiyonari H, Shioi G, Tamura A, Tsukita S (2015). Intestinal deletion of Claudin-7 enhances paracellular organic solute flux and initiates colonic inflammation in mice. Gut.

[49] Ikeda M, Fong P, Cheng J, Boletta A, Qian F, Zhang XM (2006). A regulatory role of polycystin-1 on cystic fibrosis transmembrane conductance regulator plasma membrane expression. Cell Physiol Biochem.

[50] Than BL, Linnekamp JF, Starr TK, Largaespada DA, Rod A, Zhang Y (2016). CFTR is a tumor suppressor gene in murine and human intestinal cancer. Oncogene.

[51] Scott P, Anderson K, Singhania M, Cormier R (2020). Cystic fibrosis, CFTR, and colorectal cancer. Int J Mol Sci.

[52] De Lisle RC (2014). Disrupted tight junctions in the small intestine of cystic fibrosis mice. Cell Tissue Res.

[53] Deeks ED (2016). Lumacaftor/ivacaftor: a review in cystic fibrosis. Drugs.

[54] Osawa Y, Oboki K, Imamura J, Kojika E, Hayashi Y, Hishima T (2015). Inhibition of cyclic adenosine monophosphate (cAMP)-response element-binding protein (CREB)-binding protein (CBP)/beta-catenin reduces liver fibrosis in mice. EBioMedicine.

[55] Saltz LB, Cox JV, Blanke C, Rosen LS, Fehrenbacher L, Moore MJ (2000). Irinotecan plus fluorouracil and leucovorin for metastatic colorectal cancer. Irinotecan Study Group. N Engl J Med.

[56] Seeger-Nukpezah T, Geynisman DM, Nikonova AS, Benzing T, Golemis EA (2015). The hallmarks of cancer: relevance to the pathogenesis of polycystic kidney disease. Nat Rev Nephrol.

[57] Xu C, Wang K, Ding YH, Li WJ, Ding L (2019). Claudin-7 gene knockout causes destruction of intestinal structure and animal death in mice. World J Gastroenterol.

[58] Zhu L, Han J, Li L, Wang Y, Li Y, Zhang S (2019). Claudin family participates in the pathogenesis of inflammatory Bowel diseases and colitis-associated colorectal cancer. Front Immunol.

[59] Snider AJ, Bialkowska AB, Ghaleb AM, Yang VW, Obeid LM, Hannun YA (2016). Murine model for colitis-associated cancer of the colon. Methods Mol Biol.

[60] Hu J, Harris PC (2020). Regulation of polycystin expression, maturation and trafficking. Cell Signal.

[61] Pan J, Seeger-Nukpezah T, Golemis EA (2012). The role of the cilium in normal and abnormal cell cycles: emphasis on renal cystic pathologies. Cell Mol Life Sci.

[62] Paul C, Tang R, Longobardi C, Lattanzio R, Eguether T, Turali H (2022). Loss of primary cilia promotes inflammation and carcinogenesis. EMBO Rep.

[63] Rocha C, Papon L, Cacheux W, Marques Sousa P, Lascano V, Tort O (2014). Tubulin glycylases are required for primary cilia, control of cell proliferation and tumor development in colon. EMBO J.

[64] Korobeynikov V, Deneka AY, Golemis EA (2017). Mechanisms for nonmitotic activation of Aurora-A at cilia. Biochem Soc Trans.

[65] Lal M, Song X, Pluznick JL, Di Giovanni V, Merrick DM, Rosenblum ND (2008). Polycystin-1 C-terminal tail associates with beta-catenin and inhibits canonical Wnt signaling. Hum Mol Genet.

[66] Papavassiliou KA, Zoi I, Gargalionis AN, Koutsilieris M (2019). Polycystin-1 affects cancer cell behaviour and interacts with mTOR and Jak signalling pathways in cancer cell lines. J Cell Mol Med.

[67] Happe H, van der Wal AM, Leonhard WN, Kunnen SJ, Breuning MH, de Heer E (2011). Altered Hippo signalling in polycystic kidney disease. J Pathol.

[68] Luyten A, Su X, Gondela S, Chen Y, Rompani S, Takakura A (2010). Aberrant regulation of planar cell polarity in polycystic kidney disease. J Am Soc Nephrol.

[69] Harvey KF, Zhang X, Thomas DM (2013). The Hippo pathway and human cancer. Nat Rev Cancer.

[70] Sahai E, Astsaturov I, Cukierman E, DeNardo DG, Egeblad M, Evans RM (2020). A framework for advancing our understanding of cancer-associated fibroblasts. Nat Rev Cancer.

[71] Lieu CH, Golemis EA, Serebriiskii IG, Newberg J, Hemmerich A, Connelly C (2019). Comprehensive genomic landscapes in early and later onset colorectal cancer. Clin Cancer Res.

[72] Lanktree MB, Haghighi A, Guiard E, Iliuta IA, Song X, Harris PC (2018). Prevalence estimates of polycystic kidney and liver disease by population sequencing. J Am Soc Nephrol.

