# Supplementary material for: Loss of Pkd1 limits susceptibility to colitis and colorectal cancer
Source: Oncogenesis. 2023 Aug 5;12(1):40. doi: 10.1038/s41389-023-00486-y (PMC10403611; doi:10.1038/s41389-023-00486-y)
Supplement: Supplementary file 1 — Supplemental Figures, Tables, Legends [file 41389_2023_486_MOESM1_ESM.pdf]

Supplementary Figures and Tables

Table S1. Genes with differential expression in organoids with *Apc*$^{-/-}$ versus *Apc*$^{-/-}$ *;Pkd1*$^{-/-}$ genotypes. Log2 fold change and false discovery rate (FDR) 20 (q value) are shown.

Gene name	Log 2 fold change	q-value
Krt6b	4.61854	0.00895724
Muc1	4.00522	0.0324167
Hmga1b	3.80546	0.00895724
Khdc1a	3.78241	0.00895724
Slc6a14	3.75185	0.0256887
Krt6a	3.73475	0.00895724
Slc28a3	3.67728	0.0152978
Ppbp	3.59044	0.00895724
Pigr	3.34575	0.00895724
C4bp	2.98581	0.0358289
B3galt5	2.97779	0.00895724
Krt90	2.96724	0.00895724
Cxcl5	2.9601	0.00895724
Atp6v0c-ps2	2.93357	0.0293427
C3	2.80378	0.0358289
Trf	2.74418	0.00895724
Cpne5	2.74225	0.00895724
Mrm1	2.73011	0.00895724
Cldn2	2.72018	0.00895724
F3	2.71815	0.00895724
Aldh3a1	2.65538	0.00895724
Clic6	2.64262	0.0431461
Rps2-ps10	2.60666	0.00895724
Trp53i11	2.58605	0.00895724
Krt14	2.50038	0.00895724
Tnip3	2.49658	0.00895724
Krt20	2.48354	0.00895724
St6galnac4	2.47713	0.00895724
Oit1	2.42807	0.00895724
Rps13-ps1	2.41228	0.00895724
Atp2c2	2.32493	0.0152978
Zmynd11	2.29758	0.00895724
Tgm1	2.26192	0.00895724
Atp2a3	2.23583	0.00895724
Fut9	2.22881	0.0293427
Wdr72	2.16527	0.00895724
AA467197	2.14605	0.00895724

Tm4sf1	2.14326	0.00895724
Pdzk1ip1	2.10319	0.0293427
Acsbg1	2.08097	0.0152978
Hoxb13	1.97861	0.00895724
Agr2	1.97484	0.0212734
Tmprss13	1.96575	0.0256887
Adgra2	1.95351	0.0324167
Tspan1	1.93588	0.00895724
Lypd8	1.92567	0.00895724
Hspb1	1.92512	0.00895724
Tc2n	1.91846	0.00895724
Cbr2	1.8749	0.00895724
Gabrp	1.8704	0.00895724
Ttc39a	1.8659	0.00895724
Padi4	1.84215	0.00895724
Cdo1	1.81252	0.00895724
Tfcp2l1	1.75296	0.0358289
Col4a1	1.74962	0.00895724
Pakap	1.74327	0.0212734
Col4a2	1.7392	0.00895724
Gda	1.69454	0.00895724
Naip1	1.69369	0.00895724
Pla2g4a	1.6886	0.00895724
Shf	1.6603	0.0397518
Kcne3	1.64339	0.0152978
Spp1	1.64093	0.00895724
Gk	1.62277	0.00895724
Aoc1	1.58447	0.0212734
Pdlim4	1.55867	0.0152978
Xdh	1.52945	0.0152978
Lgr5	1.48603	0.00895724
Gas6	1.46203	0.0212734
Aldh1a3	1.45895	0.00895724
Rab11fip5	1.40846	0.00895724
Ly6c1	1.40691	0.00895724
Ces2g	1.35552	0.00895724
Ctsc	1.35282	0.00895724
Rasef	1.33778	0.0256887
Acss1	1.3231	0.0293427
Basp1	1.29366	0.0152978
Ldlrad3	1.26579	0.0431461
Nectin4	1.24678	0.0152978

Gsta3	1.23209	0.00895724
Celsr1	1.2171	0.00895724
Sprr2a3	1.204	0.00895724
Sh3pxd2a	1.1936	0.0152978
Slc31a1	1.19185	0.00895724
Ckmt1	1.18173	0.00895724
Ly6d	1.14876	0.0358289
Sqor	1.13627	0.00895724
Stom	1.13178	0.0256887
Hspa1b	1.12656	0.0293427
Sat1	1.12432	0.00895724
Tmsb4x	1.12178	0.00895724
Ephb3	1.09792	0.00895724
Plac8	1.09479	0.00895724
Morc4	1.08031	0.0324167
Tnfrsf11b	1.07834	0.00895724
Wars	1.04812	0.00895724
Prss32	1.03867	0.0358289
Paics	1.03861	0.0324167
Psme2	1.03758	0.0152978
Tst	1.0288	0.00895724
Cat	1.02803	0.0256887
Cd82	1.02193	0.0324167
Rpl29	1.01064	0.00895724
Ccn2	1.0065	0.0152978
Slc12a2	0.998649	0.0256887
Clic4	0.990545	0.0293427
Maoa	0.989823	0.0293427
Wnt7b	0.986062	0.0358289
Ak2	0.983593	0.0397518
Tmem63a	0.97764	0.0256887
Tspo	0.976742	0.0293427
C130074G19Rik	0.96506	0.0358289
Ctps	0.963	0.0293427
Clu	0.96039	0.0324167
Fut8	0.959616	0.0431461
Akr1b8	0.957547	0.0324167
B3gnt3	0.932327	0.0212734
Ly6a	0.929251	0.0431461
Mmp15	0.916475	0.00895724
Sgsm3	0.915436	0.0293427
Gsta4	0.908816	0.0256887

Tspan8	0.89472	0.0212734
Emp2	0.889612	0.0397518
Gprc5a	0.888755	0.0256887
Cd44	0.876749	0.0431461
Cd9	0.831492	0.0212734
Pgd	0.803435	0.0324167
Gm8979	-1.67496	0.00895724
Igfbp4	-1.94386	0.00895724
B4galnt2	-1.97636	0.00895724
Msx1	-2.04423	0.00895724
Bex1	-2.08017	0.00895724
H2-Q10	-2.57218	0.0397518
Foxn3	-2.57239	0.00895724
Def6	-2.69764	0.0324167
Sox6	-2.80519	0.0152978
Shh	-3.22065	0.0324167
Gpc3	-5.00321	0.00895724

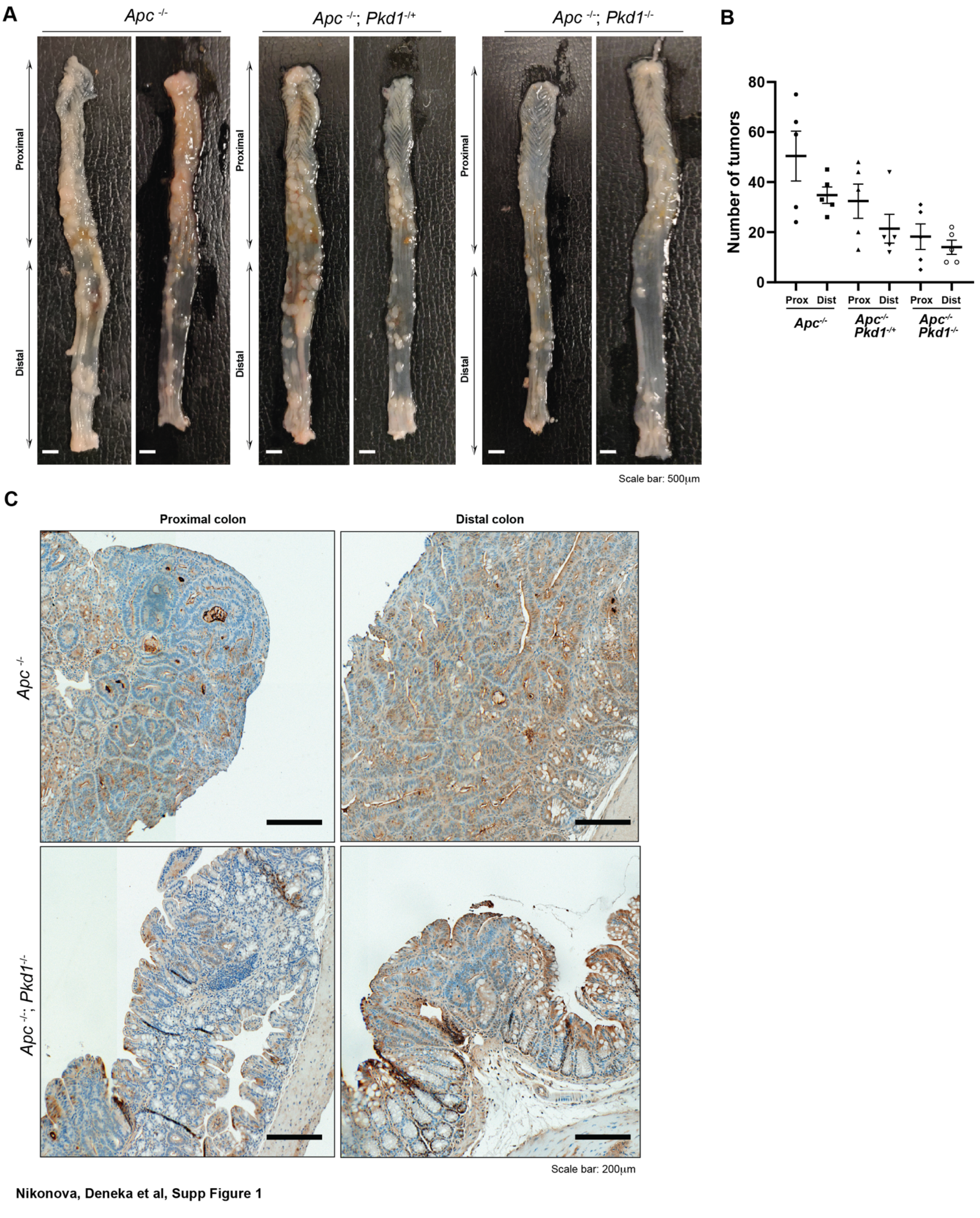

Figure S1. **Tumor number and proliferation, based on *Apc* and *Pkd1* genotype**. **A.** Additional examples of typical presentation of colons in *Apc*$^{-/-}$, *Apc*$^{-/-}$*;Pkd1*$^{+/-}$, or *Apc*$^{-/-}$*;Pkd1*$^{-/-}$ mice. Scale bar, 500 µm. **B.** Number of tumors quantified separately in proximal and distal colons of *Apc*$^{-/-}$, *Apc*$^{-/-}$*;Pkd1*$^{+/-}$, or *Apc*$^{-/-}$*;Pkd1*$^{-/-}$ mice. **C.** Microphotograph examples of Ki67 staining of proximal and distal colons of *Apc*$^{-/-}$ or *Apc*$^{-/-}$*;Pkd1*$^{-/-}$ mice. Scale bar, 200 µm.

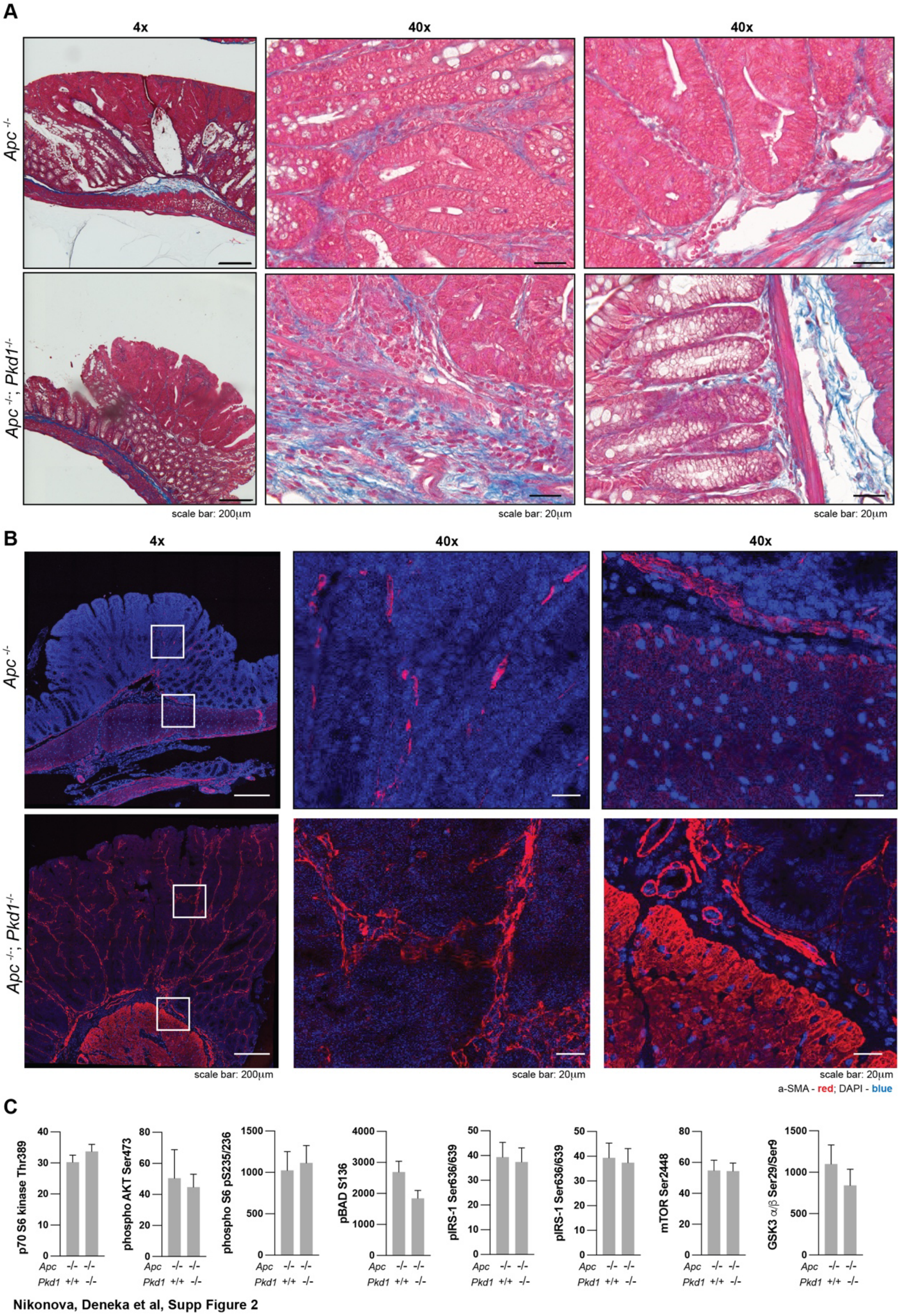

Figure S2. Fibrosis and signaling in adenomas. **A**. Photomicrograph shows representative examples of trichrome staining of colon lesions in *Apc*$^{-/-}$ or *Apc*$^{-/-}$;*Pkd1*$^{-/-}$ mice. Scale bars, 200 μm or 20 μm, as indicated. **B**. Photomicrograph shows representative examples of immunofluorescence staining of alpha-smooth muscle actin (α-SMA) staining (red) of colon lesions in *Apc*$^{-/-}$ or *Apc*$^{-/-}$;*Pkd1*$^{-/-}$ mice. Blue, DAPI staining indicates nuclei. Scale bars, 200 μm or 20 μm, as indicated. **C**. Luminex analysis of activation of proteins associated with proliferative signaling, in adenomas of genotypes listed. All results indicate non-significant differences based on genotypes.

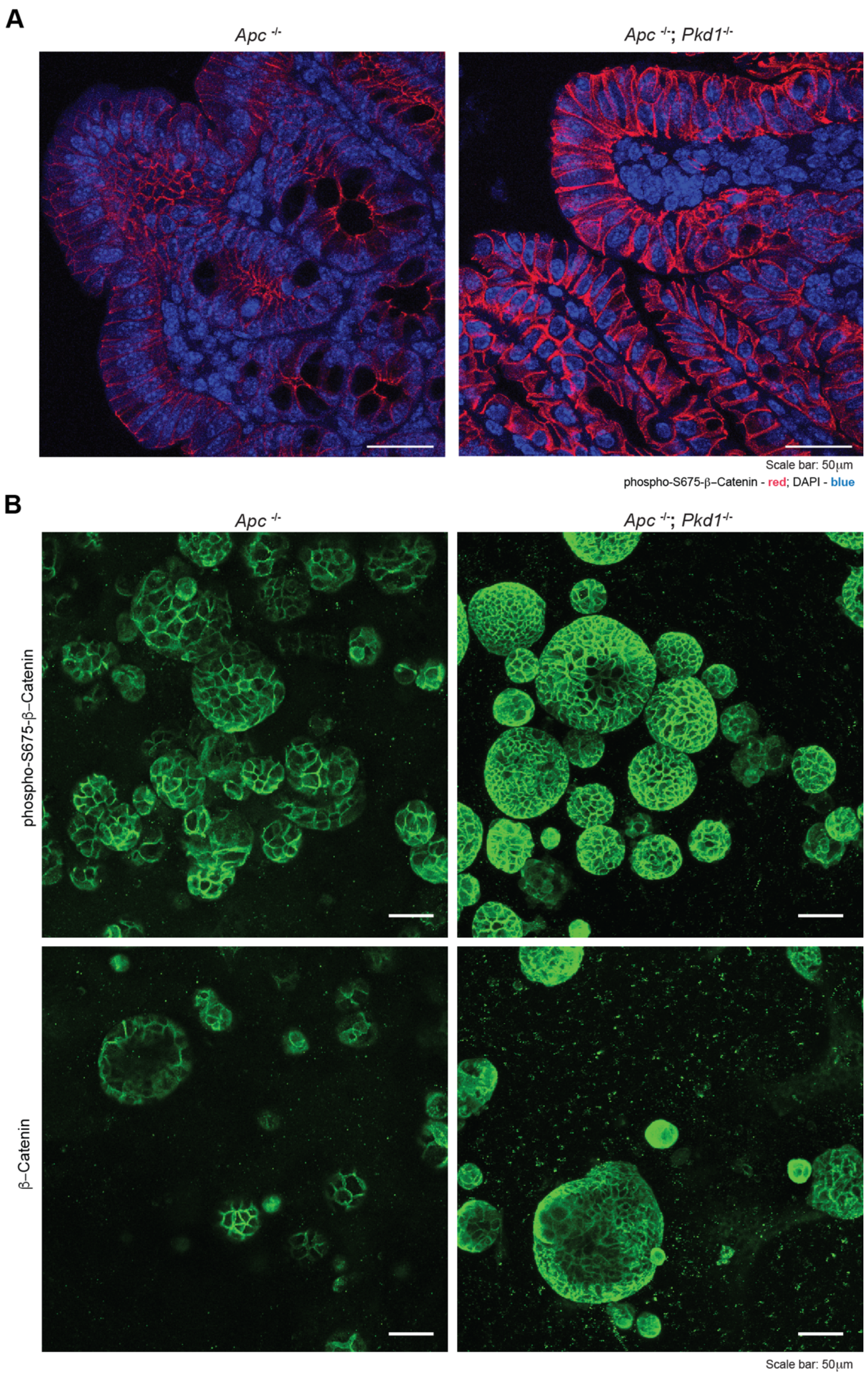

Nikonova, Deneka et al, Supp Figure 3

Figure S3. **A.** Immunofluorescence staining of lesions from *Apc*$^{-/-}$;*Pkd1*$^{-/-}$ versus *Apc*$^{-/-}$ mice colons for ph-S675-β-catenin. Blue, DAPI; red, ph-S675-β-catenin. Scale bar, 50 μM. **B.** Immunofluorescence staining of *Apc*$^{-/-}$;*Pkd1*$^{-/-}$ versus *Apc*$^{-/-}$ organoids for ph-S675-β-catenin (top) and β-catenin (bottom). Scale bar, 50 μM.

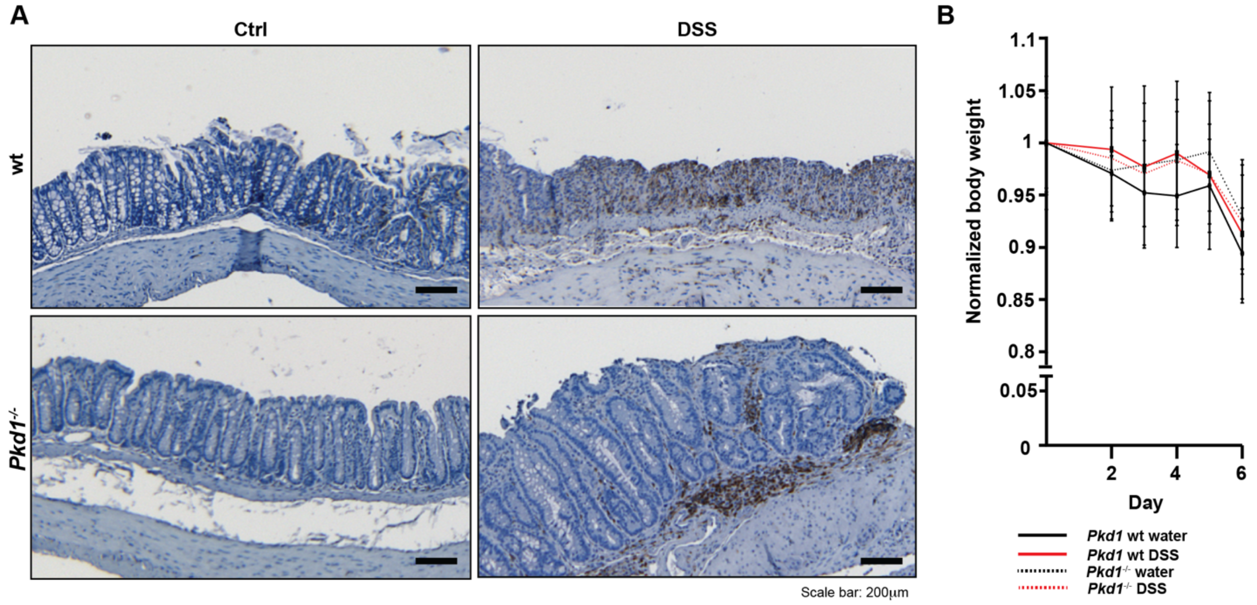

Nikonova, Deneka et al, Supp Figure 4

Figure S4. **Immune cell infiltration in DSS-treated mice, based on genotype**. **A**. Representative examples of colons from *wt* or *Pkd1*$^{-/-}$ mice treated with water (Ctrl) or DSS, stained with antibody to CD45 to visualize leukocytes. Scale bars, 200 µm. **B.** Chart representing averaged body weights of *Pkd1* ^{wt} and *Pkd1* ^{mut} mice treated with water or DSS, normalized to weight on Day 0 of the treatment.
